# Demanding stories: television coverage of sustainability, climate change and material demand

**DOI:** 10.1098/rsta.2016.0375

**Published:** 2017-05-01

**Authors:** Joe Smith

**Affiliations:** Department of Geography, The Open University, Milton Keynes, Bucks, UK

**Keywords:** television, media, climate change, material demand, sustainability, consumption

## Abstract

This paper explores the past, present and future role of broadcasting, above all via the medium of television, in shaping how societies talk, think about and act on climate change and sustainability issues. The paper explores these broad themes via a focus on the important but relatively neglected issue of material demand and opportunities for its reduction. It takes the outputs and decision-making of one of the world's most influential broadcasters, the BBC, as its primary focus. The paper considers these themes in terms of stories, touching on some of the broader societal frames of understanding into which they can be grouped. Media decision-makers and producers from a range of genres frequently return to the centrality of ‘story’ in the development, commissioning and production of an idea. With reference to specific examples of programming, and drawing on interviews with media practitioners, the paper considers the challenges of generating broadcast stories that can inspire engagement in issues around climate change, and specifically material demand. The concluding section proposes actions and approaches that might help to establish material demand reduction as a prominent way of thinking about climate change and environmental issues more widely.

This article is part of the themed issue ‘Material demand reduction’.

## 1. Introduction, methods and sources

Material demand reduction is an imperative, driven above all by the grand jeopardy of climate change. The Paris Agreement that came out of the UNFCCC COP 21 commits signatories to a cycle of emissions reductions in parallel with regular structured and transparent reviews across coming decades. Allwood *et al*. [1] have clearly demonstrated why material demand reduction is a central, but neglected, element in these processes.

They clearly establish that energy and material efficiency measures are inadequate responses on their own to the risks associated with climate change, and cannot achieve the internationally agreed policy objectives of the Paris Agreement to hold global temperature increases well below 2°C. Specifically, their work demonstrates that material demand reduction is an essential rather than desirable objective. Such findings hence need to achieve much more prominence within policy, political and public narratives about climate change. Other global environmental change issues, including biodiversity and habitat loss, and resource depletion, add further impetus to this imperative. They also note however that ‘(e)fforts … are largely stuck … . Reducing our demand for energy is essential … but is currently below the political and public horizon’ [2].

This paper explores the past, present and future role of broadcasting, above all via the medium of television, in shaping how societies talk, think about and act on issues surrounding material demand. It takes the outputs and decision-making of the UK's largest public service broadcaster, the BBC, as its primary focus. Although television outputs represent just one media form, and the BBC is just one broadcaster among many globally, there are reasons why this medium, and this broadcaster, hold particular significance in relation to global environmental change issues. The BBC's formal obligation to public service makes it a revealing case study. Its governance and funding is distinctive: it includes a near-universal requirement to pay a license fee, and is overseen by a trust, independent of government. This results in a profoundly challenging and sometimes conflicting set of obligations around diverse audience reach, popularity, comprehensiveness and accuracy in coverage, and impartiality. These obligations are summarized in a phrase coined by the institution's first director general, Lord Reith, which insists that the Corporation exists to ‘inform, educate and entertain’. The institution's distinctive governance and funding structure make it a particularly telling illustration of media engagement with a bundle of topics that generate a specific and complex cultural politics. The distinctive temporality and spatiality of climate change, and the relationships of vulnerability and responsibility that it generates pose particular challenges for storytellers in any form. Television is the predominant setting for the long-form narration of any major issue for public audiences throughout the period within which global environmental change issues have emerged. Furthermore, the BBC's formal obligations to attend to questions of civic significance *and* maintain popularity make it a powerful case study of the challenges of reaching wide audiences with ‘difficult’ issues. They are also unusual in being a global broadcaster (through World Service radio and later television channels), and for having participated in international co-production throughout the period. For all these reasons, the BBC's 50-year journey with topics that relate population, consumption, resources and environmental change make them a key reference point for any discussion of the mass-mediation of ideas around material demand reduction.

The paper draws upon material drawn from a study of 50 years of BBC broadcast archives for the AHRC funded Earth in Vision project. That project has worked with 50 hours of broadcast programming (mostly TV but including radio), totalling over 100 programmes. This body of broadcast archive has been processed in two stages: first a sample of programmes was selected to ensure a spread of content across a body of global environmental change-related themes and also channels and programme types. Second all of this material was viewed or listened to and a layer of metadata added (programme descriptions and keywords). These steps allowed for enhanced searching and relationship-building across the body of material. In the case of drafting this paper, those steps supported the identification of relevant content for consideration across the 100 programmes.

In addition to working with the archives, the research team has conducted a body of semi-structured interviews with broadcast media producers, presenters and decision-makers. The paper has also drawn upon a further set of interviews undertaken in support of reports for the International Broadcasting Trust (IBT). The 50 interviews undertaken for the IBT reports were undertaken between 2013 and 2016. The Earth in Vision interviews were fully transcribed (both the video interviews and transcripts are available publicly: see data access note). Where possible these interviews were considered in relation to specific programmes (both full programmes where available for viewing, and transcripts) and the relevant paper archives held by the BBC at their Written Archives facility at Caversham. Although the paper archives are far from comprehensive they did supply additional novel insights into production decisions.

The paper is also informed by participant observation (as academic advisor) in over 30 h of broadcast media production and is also guided by wider thinking about energy systems-transformations inspired by work on the AHRC funded Stories of Change project. It draws these materials together to respond to some of the communication, policy and political challenges raised by the discussion of material demand management by Allwood and colleagues [1,2] and responses to their work (including [3,4] and the body of papers in this special issue). It is focused upon non-news broadcasting.

The paper benefits from a body of often multi-disciplinary research that has sought to expand understanding of the varied mediations of climate change knowledge, whether through media, campaigning or the arts, including, prominently, a series of monographs by Boykoff [5], Doyle [6] and Hansen [7]. This research has also been informed by Hulme's extended consideration of the cultural politics of knowledge surrounding climate change [8] and more collective explorations of this cultural politics [9–11]. However with the exception of Doyle [6], this enterprising literature has paid relatively little attention to non-news television, and her treatment does not directly address material demand reduction. The literature on media and climate change has generated pointed critiques of both media, and research and policy community practices, but non-news television remains relatively little covered, and, within that, discussion of processes of media decision-making even less so. This paper seeks to address these gaps by bringing together a specific, significant and under-acknowledged dimension of the climate mitigation challenge, that is, material demand reduction, with analysis of relevant programmes and their production.

The first part of the paper (§2) considers some of the particular characteristics of, and hence challenges presented by, the cultural politics of climate change. Going one step further, it considers how these express themselves and are further extended in relation to material demand reduction. It also summarizes how media decision-makers respond to climate change and related themes. This section clarifies some of the underlying reasons why the cultural politics of climate change makes the production of popular television about global environmental change issues challenging. But it also notes acknowledgement by key media decision-makers that they do have a responsibility to address these themes. The following §3 reviews examples of BBC programming related to resources, population and consumption from across the last 50 years with the goal of understanding, through discussion of specific prominent examples, how particular storylines have become established and others neglected, or have been served only to specific smaller audiences.

The historical content addressed in this section was only rarely directly related to material demand and/or its reduction, but all of it connects with these themes on account of the content addressing some combination of human numbers; material consumption; and environmental impacts and changes. Section 4 introduces a body of more recent and current relevant programming that relates more directly to material demand management. Section 4b identifies some themes that could offer new directions for programming. The paper concludes with direct but pragmatic challenges to media, research and policy communities in turn. While the media are invested with a responsibility to look harder for fresh stories and approaches within these themes, research and policy participants are also pressed to become much more active in the processes of cultural entrepreneurship that will be required to place these important issues much more prominently on the ‘political and public horizon’ [2].

There is a particularly strong theme in the argument and content deployed throughout the paper: that is, that the quality of storytelling is central to the winning of a commission, the convening of an audience, and the nature of the response of that audience to these stories. One of the ambitions of this paper is to reveal the agency of the research and policy communities in generating and populating television storylines, and in so doing to encourage more direct engagement in the question of what kinds of stories television might tell next.

## 2. The cultural politics of climate change, and material demand reduction

### (a) Novel cultural politics

Climate change mitigation and adaptation present a series of challenges for media decision-makers and producers. The cultural politics of climate change can be summarized in terms of six distinctive yet often-interacting elements. These comprise global pervasiveness, far reaching uncertainty, interdependencies (both social and ecological), the reverberations of history (particularly colonial and postcolonial), the need for interdisciplinary approaches in research, and a constantly shifting distribution of human vulnerabilities and responsibilities across time and space [12]. All six dimensions are relevant in diagnosing why climate change and the consequent requirement for material demand reduction is a difficult story to tell.

One consequence of these six features is that programme makers and commissioners are challenged by the apparent lack of a human subject or protagonist, whether as victim or perpetrator in the present or future. Neither can the peculiar temporalities of climate change in general, nor material demand reduction in particular, offer any prospect of a clear denouement. When you are casting climate change who are your heroes, villains and victims? When did or does the action around a decision-with-consequences about consumption of an existing resource-hungry product or service play out: in the past, present or future? Where is the epicentre of the action: the kitchen table or the boardroom; the laboratory; the office; the street; the United Nations conference; or the presidential office? How can a programme maker turn the notion of ‘global’ into a place, except by pointing a lens through the window of the International Space Station at the ‘blue marble’ planet? Tyszczuk nicely summarizes the climate change storytellers conundrum: ‘Climate change is too here, too there, too everywhere, too weird, too much, too big, too everything. Climate change is not a story that can be told in itself, but rather, it is now the condition for any story that might be told about cities, or our inhabitation of this fractious planet’ [13, p. 47].

Additional to these six demanding characteristics, material demand reduction introduces further challenges for communicators and mediators. In the remainder of this section, I set points raised by Allwood *et al*.'s wide ranging White Paper [2] and associated publications [1,4], and in the discussion of the White Paper offered by Söderholm & Tilton [3] within the context of broadcast decision-making. These specific challenges include the challenge of telling stories about systems. They also include the fact that there are only very limited broadcast spaces available for the exploration of fundamental challenges to mainstream political economy and everyday life.

In a 2013 paper, Allwood *et al*. [14] review four main approaches to reduce emissions while meeting market demand. However challenges are noted at every turn: they note how Smil [15] summarizes the planning and regulatory tasks associated with changing energy supply infrastructures; Fouquet & Pearson [16] find that a low carbon energy transition may not deliver the kinds of benefits to producers and consumers that had promoted prior energy system transitions and Sathre & Masanet [17] review carbon capture and storage technology and lay out clearly the expense of committing perhaps one-third of the output of a fossil-fuelled power station to drive the process. There are instances where such ‘challenges’ can generate the core of a TV proposition. However, the disconnect between viewer and subject within such systems-rooted issues tends to be significant, even before the weighing of expert evidence on any particular question is considered. Hence even where the proposed strategies are impressive and wide ranging in scale, and where publics/audiences/voters will not be challenged in terms of impacts on daily life, it remains difficult for specialist factual media to derive more than an occasional story that can make it through the intensely competitive process of winning a broadcast commission.

But having noted the limits of process efficiency Allwood *et al*. [14] go further to present what is arguably an even more challenging theme for mass-audience broadcasting. They propose the need for ‘reduction in overall volumes of material production’. As they unpack their argument they present a series of often-interacting design, engineering, systems analysis, investment (corporate and consumer), trade and emissions-accounting considerations that all add up to a very complex story. Complexity is usually held to be an obstacle to broadcast media commissioning and production, Furthermore this systemic complexity is occurring within unfamiliar temporalities. These include questions such as accounting for responsibilities for historic high emissions in currently energy-efficient economies or the ‘offshored’ emissions of those same, now largely post-industrial societies. These are stories about politics. But material demand nicely illustrates the way that climate change politics resists clearly drawn territorial or temporal boundaries making it far more difficult to cast or script them.

For all these reasons to do with the systemic and complex nature of materials demand, it is difficult to shape a cohesive story that can be told within a few minutes to a peak time mid-evening audience on a popular channel. This suggests that while both policy design and media storytelling need to be informed (even if implicitly) by a more systematic approach the ‘whole system story’ will remain difficult to tell. But perhaps the most significant element of the discussion of systemic transformation required by material demand reduction is the assumption that it will require a transformation of the political economy of energy and resources. An essential step in Allwood *et al*.'s argument is that ‘the feedbacks of global warming have a relatively long time delay - and a key challenge in implementing material efficiency is to find economic justification today for actions that will benefit the population in future’ [14]. Söderholm & Tilton [3], and Low in this special issue [18], expand on this point, emphasizing the centrality of price information, particularly through taxes, in pursuing the most effective and speedy material demand reduction. Kasser [19], Whitmarsh [20], Skelton [21] and Frenken's [22] among other contributions to this special issue make clear that these economic approaches need to be placed in relation to cultural, social and political dimensions of material demand. Together these varied factors make for a rich and complex picture.

Complexity itself is not an obstacle to broadcast storytelling. However, the fact that these ideas are not circulating widely in mainstream politics and culture. Hence these dimensions of material demand reduction are perceived by media decision-makers and producers to directly challenge some stable cultural, economic and political orthodoxies. Hence media decision-makers meeting the theme of climate change in general and material demand reduction in particular are presented with actions and impacts that are complex, unsettled, diffuse in consequence and that at best imply future outcomes that are demanding and uncomfortable. This set of issues (likely to be tagged by media decision-makers as an ‘agenda’, as in ‘the green agenda’) also appear to be subversive of some central tenets of the political economy and culture of advanced capitalism regarding ‘virtuous’ relationships between economic growth, incomes, consumption, identity, welfare and social stability. Ironically, these features may help to win some slots on less-watched channels and in less-viewed slots on account of their exceptional status, and yet in turn make mainstream coverage even less likely (i.e. ‘we've done fast fashion/food waste/short life electronics already on (minority channel)’). Indeed, with the exception of some drama and comedy content, these are the only places on television that a fully argued critique of a failing system might be told from within that system.

The next sub-section sets these considerations about the nature of the issues to be explored within the context of media culture and practice.

### (b) Audiences, media decision-making and environmental change

Interviews with over 40 commissioners, channel controllers, independent producers and programme presenters from all the major broadcasters working in the UK and some North American broadcasters have presented a consistent body of challenges to telling stories about climate change on television. Many of the arguments made in these interviews map closely onto the points noted in the previous section regarding the cultural politics of climate change, but place them within the cultures and practices of broadcast decision-making. Taken together, the interviews suggest that media decision-makers consistently believe that global environmental change-related programming tends to be difficult to commission, make and, crucially, watch.

They are concerned about what audiences will ‘do’ with this difficult new knowledge and fear the disempowerment that comes from an over-familiar litany of doom-mongering and demands for denial. They also doubt that audiences will come, or will stay watching, most environmental change-related material. The majority of the programmes that address climate change do not rate well except those that take a counter-cultural point of view. One reason identified is that the very nature of the topic means that it is difficult to satisfy the need for resolution within the life of an individual programme or series. Respondents consistently returned to questions of tone too: they insisted that (audiences) do not want to be preached at or blamed. It was also felt that the topic had lost its capacity to shock.

When asked about what solutions might exist in terms of bringing evidently important themes to television there are again some consistent responses, above all noting the centrality of the quality of storytelling, and of relating abstract issues to concerns in everyday life, above all household or family life. Given the familiarity of the topic channel controllers and commissioners in particular have emphasized the importance of fresh approaches. Talking of specialist factual, such as lifestyle or home improvement programming, producers insisted that the offer to audiences needed to be aspirational and on-trend. There was also regular reference to the need to make full use of the potential of the ‘second screen’, i.e. the social and digital media offer associated with broadcast programming. Online content by its nature can afford to reach more tightly (often self-) defined audiences, and also opens up potential for more participatory media.

These challenges and responses are not all shaped by the particular characteristics of environmental issues: they are also a measure of far-reaching changes in media economics, production, consumption and in some cases form. This creates some new opportunities to serve ‘committed’ and engaged audiences even better (i.e. the ‘citizen channel controller’ who can devise their own schedule of ‘improving’ content). But these developments also make it less likely that mass audiences will make appointments to view environmental change-driven programming (i.e. consumer schedulers, who can exclude unwelcome or challenging content and stick with comforting favourites). Interviews suggest that, with only a few exceptions, media decision-makers view approaches that implore reduced consumption and personal denial to be ‘ratings toxic’.

The figure of the audience is foremost in the media decision-maker's mind at every step, including ideas development, pitching, commissioning, production (including editing in response to feedback, for example, from channel and co-producer executives), and assessment of reception. But what this concern for audience response means in practice varies widely. Even in simpler times when television was the only screen in the room, media decision-making did not consider audiences to be one undifferentiated mass. A channel controller and scheduler, and genre-specific commissioners, carry a clearly defined notion of the demographic of their channel. These directly shape what viewers might encounter on a particular channel at a particular time. Hence a Friday evening slot on a popular channel will draw an audience of several million that anticipates relaxation or escape. A minority specialist factual channel might use a late-night slot to satisfy a discrete set of tastes and expectations about challenging factual content for an audience of 100 000. These often fine-grained distinctions between channels, and between slots on channels, have been complicated but not much eroded by the emergence of new ways of watching TV presented by set-top boxes and video on demand services on a wide range of devices and platforms.

The next section reviews a body of examples of programming spread across 50 years. It explores how population and end-of-process concerns with pollution became dominant storylines rather than *per capita* consumption of materials; how reduced consumption themes have on occasion won airtime, but primarily by being framed as exotic and alternative. The section goes on to consider how more recently there have been successful programmes (in terms of both viewing figures and peer critique) that have addressed dimensions of material demand reduction.

## 3. Fifty years of broadcasting about population, resources and environmental change

### (a) Why population and pollution, not consumption?

The relationship between resources, population and environmental degradation has a long history within broadcasting, and material demand is a discernible strand within it, although there is a consistent trend that sees population—sheer human numbers—identified as the driving problem, rather than the *per capita* growth in the developed world's material or wider resources demand. Understanding the origins and dynamics of this in broadcasting in the past by looking in more detail at a small number of examples helps in considering routes to reframings of material demand reduction in the present.

One prominent Western political and cultural frame throughout the second half of the twentieth century was of an endlessly growing economy, allied to the notion that both cultural and material rewards were more widely and evenly distributed than ever before. In the context of the Cold War, this frame had a more or less explicitly ideological relevance in the West. Hence it is more than an aside to note that what environmental historians and scientists have dubbed the ‘great acceleration’ [23] that transformed Western economies and societies was not just televised: it made television the culturally powerful medium that it is today. The explosive expansion of TV set ownership, the advertising industry, the advent of colour television and the increase of material demand in the second half of the twentieth century were all closely interrelated. The travel and home improvement strands of the emergent genre of factual entertainment were prominent examples of the ways in which hydrocarbons, technology and increased earnings across the middle and working classes were combining to offer new freedoms, life choices and experiences. Documentary and factual magazine shows followed the changing nature of technology, work, community and home life. They were frequently presented with a celebratory tone. This found echoes in aspirational dimensions of sitcoms and dramas. Satellites and the space race both enabled and represented a globally distributed sense of exhilaration at technical, and implicity social, progress. This was nicely expressed in the first live global satellite-enabled broadcast *Our World*, of 25 June 1969, which included a premiere live rendition by the Beatles of *All You Need is Love*.

However, the notion of a broadcast media-generated sense of a shared ‘global’ identity was quickly joined by more difficult knowledge: that is, of the possibility that our planetary home was threatened by humanity itself. A parallel and critical set of storylines came to prominence in the 1960s and early 1970s concerning resource crises, population and threats to both civilization and ‘nature’. The specifically environmental hazards and losses generated by the great acceleration were explored, mostly on less popular channels or in less popular time slots, although a sprinkling of special feature documentaries received more prominence. Within public service broadcasting, these issues tended to be thought about in commissioning and programming terms as a form of ‘minority report’ that might be offered occasionally within flagship documentary strands, or given space on BBC2 (launched in 1964), which was not burdened as BBC1 was with the expectation of ‘winning the evening’ in ratings terms from commercial rival ITV. Such coverage was a way of respecting obligations to ‘educate and inform’ regarding emerging or prominent bodies of thought while protecting audience share elsewhere on the network.

But population and resources stories could satisfy some of the expectations of broadcast decision-makers. The intense and declensionist tenor of these kinds of arguments was attractive to commissioners on account of their radical and ‘dissident’ status. Declensionism, in reference to both environmentalism and environmental history and related research, is ‘a process by which a reasonably beneficial environmental situation became progressively worse due to human actions’ [24]. These arguments made interesting broadcasting, worthy of scarce airtime, on account of their extreme contrast with the core storylines of post-war broadcasting about the economy, society and the future.

The critical approaches tended to be informed implicitly or explicitly by neo-Malthusianism. *The Challenge of the 60s* refreshed the arguments of eighteenth-century economist and Anglican priest Thomas Malthus, and more recent work of economists and geographers such as the American scholar William Vogt. Malthus had argued ‘ … the power of population is indefinitely greater than the power in the earth to produce subsistence for man’ [25]. Vogt's 1948 book *The road to survival* is largely forgotten today, but it had reached 20–30 million readers with a very similar line of argument [26]. For Malthus, Vogt and the American academic Paul Ehrlich, author of the influential 1968 bestseller *The population bomb* [27] if population growth outstrips food supply the inevitable outcome is famine and conflict—a ‘Malthusian crisis’. The BBC regularly returned to the theme of population, generally running with the ‘population bomb’ line of argument where increasing human numbers were viewed as a threat with only sparse references to levels of *per capita* consumption.

In one episode of a 1967 BBC2 series on *The Population Problem*, a Prof. Fremlin predicts in a studio discussion that ‘in the future we'll all be living underground eating minute floating plants’. The expert suggests that on account of population growth we will in future all have to live underground, or in underwater cities, so that all productive land and water can be put to work using sunlight to produce food. ‘Is there an even more drastic stage?’ asks the deadpan presenter Derek Cooper. ‘Yes’ replies Prof. J. H. Fremlin, with a twinkle in his eye. ‘If we begin to synthesise our food chemically… Using as our raw materials mainly human excreta with a little bit of addition of assorted minerals. I don't think we'd need to feed the bodies back in. I think we could do without that. Then one could go on producing as much food as we needed for as far as one can see’.

These accounts of the relationship among population, resources and environmental change predated the formation of some of today's most influential environmental NGOs, and were prominent features in the cultural landscape of their foundation. Notable among these broadcast projects is British ecologist Frank Fraser Darling's BBC Radio 4 Reith lectures of 1969, entitled *Wilderness and Plenty*. Files in the BBC's paper archive held at Caversham show that selection of the lecturer for this high profile lecture series for the main speech radio station in the UK was carefully considered. The process of commissioning included a significant degree of prior editorial shaping of the theme that might be addressed by BBC staff, even before he was shortlisted. A lengthy memo from the Head of Talks and Current Affairs (Radio) opens with the statement that

The search for the significant and journalistically apposite theme, combined with a speaker of appropriate stature and ability, has proved no easier this year than any other … It would seem to me that what we need is a scientific approach, intensely aware of social consequences, to some imminent major change or danger likely profoundly to affect mankind.

It reviews two other possibilities before suggesting that

‘it could be (c) ‘Waste, Want and Wilderness’ – in effect a warning about the ecological and environmental changes now being brought about haphazardly and with uncontrolled and gathering momentum’ (J.A. Camacho to MDR, 1 April 1969)

In correspondence during the consideration of Frank Fraser Darling, the Editor of Science Talks invites him to lunch to discuss potential programming given the fact that ‘We have noticed that 1970 is European Conservation year, etc., etc., and of course, there was the OECD inter-governmental conference in Paris in September. This has set us thinking afresh and we are interested not just in conservation with a little or even a big C, but the future of the biosphere and man in it’ (Correspondence: Archie Clow to Frank Fraser Darling, 20 February 1969). In the wake of the lunch Fraser Darling outlines the route his Reith lectures could take, arguing that ‘we desperately need to tackle human problems ecologically — the term now is the “ecosystem approach”! Further I have a beautiful lot of stuff on lop-sided development and the consequences’ (23 March 1969 Correspondence, FFD to Dr Archie Clow). His pitch asked: ‘How do we arrive at a modus vivendi which will not render civilization a contradiction in terms ecologically?’ (Frank Fraser Darling proposal presented to Archie Clow 23 March 1969 BBC).

In the course of the lectures, he acknowledged his intellectual debts to Malthus, Darwin and ‘the Gloomy Dean’ of St Paul's Cathedral, Dean William Ralph Inge's 1920s writings on, among other things, eugenics. In his opening radio lecture in the series Fraser Darling noted how ‘governments and United Nations agencies are feverishly carving up the remnants of nature's wilderness wherever it is thought possible to grow more food or hold more water for the increasingly articulate hungry millions’.

The energy crises of the early 1970s were driven by geopolitical rather than physical resource constraints but they appeared to confirm the neo-Malthusianism of Darling's lectures and earlier coverage such as the Panorama documentary strand specials *The Challenge of the 60s*. These events served to renew broadcaster appetites to engage with the issues around human numbers and environmental hazard, but the relatively rare explorations of *per capita* consumption focused not on the increasing intensity of the mainstream, but rather exotic and scarce examples of its opposite.

### (b) Alternative spectacles

While the nexus of population, resources and pollution formed the core of factual coverage of emergent global environmental change issues these were translated into specific themes and treatments in novel factual entertainment formats. These represented responses to environmental issues within households and individual life stories. Programming on self-sufficiency and alternative technology gained a place in TV schedules, tracing purposeful decisions to reduce an individual's or a community's demand for materials and energy. These were portrayed as exotic, eccentric and exceptional. These, after all, were the characteristics that might get a programme commissioned, aired and watched. Hence just as with the more macro-coverage of population and resources issues these ‘alternatives’ were presented as an outlying critique of the strongest currents of mainstream culture and political economy, albeit at a far more intimate scale.

Programming on the theme of self-sufficiency is exemplified by the regular media appearances of John Seymour. Seymour's 1976 volume [28] on self-sufficiency was considered the bible of these practices. A 1975 BBC2 feature documentary *Living On the Land: Self-Sufficiency at Fachongle Isaf* depicted life on his Welsh smallholding. This setting provided a telegenic platform for his proposal that: ‘A widespread return to peasant farming is desirable and may well become a necessity’. Reflecting on why he believes vegetarianism to be environmentally damaging he adds that unchecked populations ‘will proliferate and die of starvation’.

The same themes of self-sufficiency, simple living and alternative community were also explored in episodes of *By Way of Change*, a documentary series which explored radical living experiments, including *The Shrub Family* (episode 6). The Shrub Family intentional community, or commune, sought to reduce their impact on the natural world through sharing accommodation and other goods. In one filmed sequence, one couple share a bath while two other members of the community chat with them, one perches near the bath reading aloud a letter to the community from a shared friend. The film-makers tone and gently paced approach is sympathetic. The channel controller could be confident that the to-most bizarre spectacle of shared baths, houses and jobs would draw a decent audience.

In an early example of what has come to be known as reality television a 1975 film in *The World About Us* documentary strand *Back to Nature* followed a group of volunteers as they lived off the land in a remote part of Exmoor, southwest England, for a fortnight with just one set of clothes each, knives, string and one bag of oats. These first examples of the reality TV genre on British television are organized around the idea of giving up material goods and modern living. Explicit reference to simplicity and a rejection of material consumption within settings that are clearly exceptional provides programme makers with interesting and watchable forms of difference, both in terms of what's said and what is seen. Challenge elements serve to generate peaks of jeopardy for many of these otherwise slow-paced films. In the closing lines of *Back to Nature*, the voiceover, as they walk off the moor at the end of their fortnight states that ‘we had proved something to ourselves, if only that there are many things in life that we can do without. At the same time we are filled with an unholy joy at the prospect ahead of us of the lonely cafe…’

The influence of this coverage of alternative simple living upon mainstream broadcasting was confirmed more than anything else by the commissioning of the sitcom *The Good Life.* Two strongly contrasting patterns of material consumption pursued in adjacent suburban households provided the narrative spine of this popular sitcom. Whether in sitcoms, proto-reality television or documentaries, this coverage of self-sufficiency and alternative living experiments gave commissioners and viewers characters and settings that were exotic but that also referenced, in romantic light, simpler and more cohesive past times.

As with the representations of the population crisis storylines, self-sufficiency television was commissioned on account of its exceptional status rather than having the goal of encouraging reductions in material demand. But programmes such as these showed that innovation in broadcast storytelling, and in theme, was possible and could draw an audience, and in doing so seed questions about quality of life and material consumption. Both *By Way of Change* and *Back to Nature* were produced by anthropology graduate John Percival, who also produced another innovative and exceptional (in the sense of rare) strand in the form of the first environment themed UK-produced TV magazine show *Down to Earth*. Like *Nature,* the BBC2 30 min documentary strand that followed in the 1980s (running from 1983 to 1994), it had a markedly journalistic and investigative purpose that distinguished it from most other nature and ‘environment’ programming.

A more mainstream treatment of energy and resource demand management is offered in a BBC1 documentary series *What on Earth… are we doing?* The episode *Power to the People* presented by broadcaster and naturalist David Bellamy is unusual in consistently drawing critical attention to the extravagant energy demands of modern life, while acknowledging its pleasures. Within the title sequence, the Earth is viewed from space, and a voiceover notes ‘Man launches himself into space and for the first time in history can see himself and his world’. The programme's theme and tone however is down to earth: the opening scene tours the English seaside resort of Blackpool's extravagant illuminations. It moves into a collage of: the town's famous light display; one arm bandit gambling machine and newspaper headlines, settling on the words ‘Fuel Crisis Crunch’.

The presenter opens with the statement: ‘Blackpool at night. It doesn't look as if the end of anything is nigh let alone the end of the world's energy supply’. The script includes lines that appear to come directly from the environmental economics literature of the period [29,30], for example, coal is ‘the capital of the earth that we are rapidly using up’. Concluding a tour of the new coal fired power station at Ratcliffe on Soar, including a nod to its innovative and award winning designs and efforts to satisfy environmentalists through monitoring and other measures, the script notes that ‘these sophisticated structures are also symbols of the urgent need for power conservation. We are wasting more energy than we put to good use’. By the way of contrast, the programme then moves to the urban self-sufficiency experimental community at Street Farm. This anarchist collective, inspired in part by situationist thinking [31], was a lived example of both political and technological ideas circulating in the alternative technology movement. The voiceover notes that ‘Graham (Caine) and his revolutionary friends are only part of the wider experiment in alternative technology. Science can offer economical alternatives that make less demands on resources, like this simple heat exchanger that draws more heat from the atmosphere outside than from the energy in fossil fuels… We don't have to go right back to nature but we might stop trying to dominate her’.

The tone was driven by the trademark approachability and winningly direct and conversational manner of this popular presenter. This combined with a mix of household consumption settings and established and ‘alternative’ technology to provoke audiences with a clear but engagingly presented message. This example nicely corresponds with many of the conclusions in the contemporary literature on media, climate change and social action (e.g. [5,6,8,9]) by clearly locating risks and actions within everyday life, and in an empowering tone. The editorial framework would need relatively little amendment to feel timely today, with its call for reduced energy and material demand via consumer, technological and institutional responses. But then as now, such an appeal was exceptional, occasional and despite the creative investment, would probably have sat awkwardly in the schedules.

## 4. Twenty-first-century consumption in the frame

### (a) ‘It is not easy’: recent programming addressing consumption

Tone remains a central concern for media decision-makers who seek to connect audiences to the environmental consequences of contemporary life. In a substantial body of interviews with over 50 media decision-makers there were regular references to a small body of shows deemed successful both in ratings terms and in respect of their engagement with environmental change themes. One example of successful character-driven demand reduction television is the BBC2 show *It's Not Easy Being Green*, which ran for three series, broadcast in 2006, 2007 and 2009). Its success can in large part be measured by the simple fact that it was twice re-commissioned. Although it carried some of the motifs of the ‘alternative lifestyles’ documentaries of the 1970s, it was produced with upbeat graphics and music, and relentlessly positive central characters. The concept was developed and pitched within the idiom of home improvement and building shows. The demands required by the larger environmental impact-reduction measures however did tend to confirm the somewhat apologetic title, and a range of difficult questions about material demand went under- or unexplored (including rebound effects of financial savings, or complexities of environmental impacts of reuse [32,33]).

The long-running (since 1999) Channel 4 home-building series *Grand Designs* was also offered by several media decision-makers as an example of sustainability thinking, again set within the idiom of aspirational lifestyle television. However, this aspirational specialist factual programme carries more content about consuming ‘well’ than less. Perhaps in acknowledgement of this, the on-screen talent Kevin McCloud has also presented series and one-off shows devised around or linked to material demand reduction, including *Kevin McCloud's Man Made Home* (Channel 4, 2012 and 2013) and *Kevin's Supersized Salvage* (Channel 4, 2014) that several interviewees offered as examples of taking sustainability into the heart of mainstream television schedules.

There is also evidence of the importance of entrepreneurship by programme makers and their presenters and advisors, in other words of taking financial or creative risks in order to present important but ‘difficult’, ‘invisible’ or abstract issues in new ways. The passive ignorance of energy systems and costs provided the device behind the 2009 BBC1 science magazine show *Bang Goes the Theory*'s one-off special *The Human Power Station*: ‘We're going to put a typical family's energy use under the spotlight to examine our love of this stuff: electricity’. Presented as a ‘massive experiment’ the peaks of jeopardy were generated by whether the volunteer family's ordinary routine could be powered by the 80 cyclists who were hooked up to generators and concealed in the vast studio next door. Climate change was not referenced within the programme, rather it was explained in terms of the fact that ‘we're told we face a global energy crisis which means big price hikes and worse still shortages. So it's a good time to ask: how much power do we all use, where do we waste it and can we do anything about it?’ Listing examples of standard household energy saving advice the script suggests that ‘ … you could save yourself quite a bit of cash, and probably, reduce the damage to the environment at the same time’.

In terms of material demand reduction, the programme was notable for its critical focus on household appliances. While the show focused on their energy consumption it did graphically represent, and emphasize within the script, the rapid increase in the scale and range of types of appliances owned in recent decades. But the stunt nature of the programme, with its big reveal for the volunteer family who had gone about their daily lives ignorant of the surges of peddling that casual events in their daily routines had required, is not quickly repeatable. It was also an expensive show. *The Human Power Station* also demonstrated the point made by several media decision-makers that these issues require ‘big ideas’ and ‘heft’ if they are to be commissioned and then break through to increasingly distributed public attention.

Similar entrepreneurship was demonstrated in the BBC3 commission *Blood Sweat and T Shirts.* This was a BBC3 (youth/young adult audience) commission that won both strong audiences and critical acclaim for its simple but fresh approach to confronting consumers with the environmental and social costs of their purchasing decisions. This blend of fly-on-the-wall documentary and travelogue set critical and provocative questions within shows that offered viewers engaging personalities and novel settings and stories. It is perhaps the best example of television that addresses Fletcher's call for a contrast to be drawn between fast fashion and ‘emotionally durable’ garments [34]. Later commissions ‘ … and Takeaways’, ‘ … and Luxuries’ further confirmed the value of an approach that deftly achieved the ‘intertwining of ethical and emotional responses’ noted in Harrod's discussion of Ruskin [35].

One other recent pair of series drew positive comment from media decision-makers in that they demonstrated that it was possible to make successful television around issues of waste. *Hugh's Fish Fight* (Channel 4, 2013) and *Hugh's War on Waste* (BBC1, 2016) were considered outstanding examples of ‘campaigns in a features wrapper’ by one TV commissioner. In this case, the presenter had strong authority on environment-related topics with the audience, hence meeting the requirements of effective celebrity leadership [36]. But the campaign-driven format was risky, and the underlying, long-standing issues underpinning the programmes (fishery discards; waste streams) were at first sight complex and dull. Notably, the digital strategy for the programmes succeeded in further activating public engagement. It pressed for, and tracked and publicized, actions by the institutions targeted for their wastefulness. It is significant that the marketing and editorial scaffolding of these successful programmes, and their associated online extensions and policy campaigns, was driven more by collective affront at the waste, rather than abstract appeals to the protection of the interests of the non-human natural world, or younger or future generations.

### (b) Stories for the future

Some of the most pressing jeopardies facing humanity are at first glance unattractive topics for media decision-makers both because they view them as ‘difficult’ stories to tell, and they believe audiences will not come to watch them. While this is true of climate change and biodiversity loss in general the arguments put for significant material demand reduction by Allwood *et al*. [2] are at first sight even more difficult propositions. On top of all of the challenges that come with the distinctive cultural politics of climate change, arguments for substantial material demand reduction bring additional quandries. They are rooted in fine grained and shifting technical knowledge about the which, how, where and when of material demand reduction (see, for example, [32] or [21] for reviews that reflect this complexity). A deeper buried, but perhaps more significant obstacle is that the goal appears to question some deeply entrenched assumptions about interrelations between the economy and social and cultural stability. The unwritten social contract of late capitalism assumes ever-expanding and/or sustained high levels of material consumption as a route to fulfilment and self-expression. This has proved a very robust frame, and despite very varied efforts across several decades has been not been supplanted by competing frames rooted in arguments about environmental costs of, or limits to, human development.

What opportunities exist to challenge those assumptions within the context of television production and reach mass audiences with a different set of proposals about our life with stuff? Following the advice offered by commissioners and producers, the first task is to consider whether there are *human* stories to be told. On close inspection, the signs are encouraging. Varied disciplinary contributions to an interdisciplinary workshop exploring routes to material demand reduction offered a range of proposals that could promote action [4]. These included aligning deep and widely held values, such as treasuring time spent with loved ones, with consumption; learning from the achievements of health and safety legislation including asbestos, seat belts and toxic dumping action; bringing together health and climate goals, for example, around diet; focusing on the highest climate impact actions not simple consumption, while keeping a close eye on inequality of impacts; making sure energy is used where it is most highly valued, i.e. get the price right, and sharing more vivid and concrete visions of the low carbon life and promoting the improvements in everyday quality of life and work.

None of these are novel insights, and some have featured in news and non-news broadcast content. Most of these can be directly connected—some powerfully—to located and familiar everyday concerns, and hence answer varied calls coming from social and cultural analysis regarding the framing and content of climate communications (including [5,6,8,37–39]). Stories of health, work, home life, food and feelings are fundamental to everyone. However, critiques or commentaries on the downsides of consumption tend to have the feel of ‘minority reports’ or ‘faults that need fixes’ on a small scale, and do not accumulate to shape an alternative, and equally influential frame to the persistent signals that affirm the aspiration for, or experience of, high-consumption economies and lifestyles.

## 5. Conclusion

Where might compelling stories about material demand reduction come from? How might these aggregate to offer a reframing of the relationship between economic and social life and material demand? It is essential that our lives with stuff, with things, are represented and talked about in the mass media—above all in television—in ways that support rather than impede transitions to more careful ways of thinking about materials.

The paper has outlined how the challenge set by Allwood *et al*.'s White Paper [2] and associated publications has to be addressed with an understanding of the very particular cultural politics surrounding climate change and sustainability issues. It has noted the centrality of the figure of the audience in media decision-making, and of effective television story telling in gaining and keeping an audience for a particular programme on a particular channel in an increasingly competitive environment. The paper has offered an historical account of how population, resources and environment issues have been represented on BBC television, and considered recent examples of programming that has engaged more directly with material demand reduction. It has plotted the ways in which some notable recent achievements in terms of strong audiences and critical reception have been arrived at by embedding ‘stories about stuff’ within established popular shows and formats.

The first requirement for more impactful storytelling about climate change is a spirit of creative entrepreneurship. New phrases, images and arguments (or repurposing of old ones) are required. And a testing of these—sometimes rigorous, and sometimes playful and experimental, will be needed too. It is helpful to note that this kind of productive interaction between research, media and policy communities has been seen before in response to difficult new knowledge around global environmental change. Ideas such as spaceship earth; limits to growth; biodiversity (loss); ecological footprint, the population explosion and most recently the anthropocene were generated as phrases that were shaped to be shared. They were intended not for the seminar room, but to do work in the world. Some stand up better to interrogation than others, and some, arguably, have done more damage than good in terms of advancing well-considered and purposeful action. But they do all serve as a reminder that the research and policy worlds have the capacity to be intellectual entrepreneurs ‘out in the world’. Such collaboration can help to unsettle and question currently predominant narratives that imply a lock-in to high material consumption societies.

But above all television needs good stories. Definitions of a ‘good story’ vary but television executives have three proxies: strong ratings, positive critical reaction and a less tangible sense of whether or not a programme has been ‘talked about’ (whether within the industry or more widely in society). For anyone in the policy or research communities concerned with material demand reduction a good story will also need to support macro or micro changes to systems and/or everyday lives.

To help this search for powerful phrases and tones here, by way of an example, is one simple proposal: to focus on the word quality. This special issue demonstrates how well founded critiques of current systems of material demand can come from many quarters: from design, the social sciences, economics, engineering or in other registers, from philosophy or theology. Quality is one potential unifying theme. In other words, pursuit of demand reduction should go hand in hand with a purposeful revision of the public and private sense of the meaning of the word ‘quality’. The papers presented here make very clear why the policy and research communities concerned to progress action on climate change need to pay much more prominent attention to material demand reduction. But for various reasons this argument is in danger of being seen as an addendum to the ‘main action’ of attempts to reduce ‘end of pipe’ emissions reductions, and hence a task that is destined to be neglected.

Researchers can apply forensic rigour to the question as to whether a steel supply and specification system or a coffee cup has ‘quality’ in terms of its contribution to our individual and collective futures. This will help to embed potentially engaging stories within more or less everything that people look at, touch and use. Thus, the stuff society lives with might gather an association with having an inner beauty or inherent ugliness. In this way, researchers can play a strategic role in shaping the extent to which a product or service tells a ‘good-quality’ story. In so doing they will equip television producers with the raw materials for scripts and filmed sequences that can quietly but purposefully revise notions of a ‘good life’.

Creative and entrepreneurial partnerships between researchers and media professionals could thus catalyse broader consideration of and response to good and bad qualities. This is not an entirely novel idea: a number of the broadcasts reviewed in this paper have touched upon such an approach and been deemed successful in both creative and communication terms. But these currently amount to sparse rehearsals rather than a sustained re-framing of the relationship between environmental change, material consumption and everyday life. However, they do on occasions show how rather than being associated with sacrifice and denial such approaches can redefine what it is to have a good life [40]. That is the point at which stories start to travel and be shared widely, and do their own work in the world.

## Data accessibility

Programme clips, broadcast archive materials, interviews, transcripts and three e-Books relating to the AHRC funded Earth in Vision project are freely available at www.earthinvision.org. Interviews and transcripts relating to the AHRC funded Stories of Change project are also available at www.storiesofchange.ac.uk. The paper archive that informed the Earth in Vision project was the BBC Written Archives Centre at Caversham. It is a resource available to researchers by appointment. Contact: heritage@bbc.co.uk; BBC Written Archives Centre, Peppard Road, Caversham Park, Reading, RG4 8TZ, UK. Phone: 0118-948-6281.
